# Exploring Periodontal Conditions, Salivary Markers, and Systemic Inflammation in Patients with Cardiovascular Diseases

**DOI:** 10.3390/biomedicines12061341

**Published:** 2024-06-17

**Authors:** Carmen Silvia Caloian, Petra Șurlin, Andreea Ciurea, Dana Pop, Bogdan Caloian, Daniel Corneliu Leucuța, Adrian Bogdan Țigu, Giulio Rasperini, Iulia Cristina Micu, Alina Stanomir, Andrada Soancă, Alexandra Roman

**Affiliations:** 1 Department of Periodontology, Faculty of Dental Medicine, Iuliu Hatieganu University of Medicine and Pharmacy, 400012 Cluj-Napoca, Romania; caloiancarmen@yahoo.com (C.S.C.); andreea_candea@yahoo.com (A.C.); i.cristina.micu@gmail.com (I.C.M.); alina.stanomir@yahoo.com (A.S.); veve_alexandra@yahoo.com (A.R.); 2 Department of Periodontology, Faculty of Dental Medicine, University of Medicine and Pharmacy Craiova, 200349 Craiova, Romania; surlinpetra@gmail.com; 3 Emergency County Clinical Hospital Cluj, 400006 Cluj-Napoca, Romania; pop67dana@gmail.com; 4 Department of Cardiology-Rehabilitation, Faculty of Medicine, Iuliu Hatieganu University of Medicine and Pharmacy, 4000347 Cluj-Napoca, Romania; 5 Department of Medical Informatics and Biostatistics, Faculty of Medicine, Iuliu Hatieganu University of Medicine and Pharmacy, 400347 Cluj-Napoca, Romania; danny.ldc@gmail.com; 6 Research Centre for Advanced Medicine (MEDFUTURE), Iuliu Hatieganu University of Medicine and Pharmacy, 400347 Cluj-Napoca, Romania; adrianbogdantigu@gmail.com; 7 Department of Biomedical, Surgical and Dental Sciences, University of Milan, Foundation IRCCS Ca’ Granda Policlinic, Via della Commenda 12, 20122 Milan, Italy; giulio.rasperini@unimi.it

**Keywords:** periodontitis, atherosclerotic cardiovascular disease, atherosclerosis, atrial fibrillation, S100A8/A9, kallikrein

## Abstract

(1) Background: This cross-sectional investigation appreciated the role of serum C-reactive protein (CRP), several hematologic-cell markers, and salivary inflammation-related molecules [calprotectin (S100A8/A9), interleukin-1β (IL-1β), kallikrein] to predict periodontitis in patients with atherosclerotic cardiovascular disease (ACVD), arrhythmia, or both. Also, we appreciated the relationship between the inflammatory burden and periodontal destruction with the type of cardiac pathology. (2) Methods: Demographic, behavioral characteristics, periodontal indicators, blood parameters, and saliva samples were collected. (3) Results: All 148 patients exhibited stage II or III/IV periodontitis. Stage III/IV cases exhibited significantly increased S100A8/A9 levels (*p* = 0.004). A positive correlation between S100A8/A9 and IL-1β [0.35 (<0.001)], kallikrein [0.55 (<0.001)], and CRP [0.28 (<0.001)] was observed. Patients with complex cardiac involvement had a significantly higher number of sites with attachment loss ≥ 5 mm [19 (3–30)] compared to individuals with only arrhythmia [9 (3.25–18)] or ACVD [5 (1–12)] [0.048♦ {0.162/0.496/0.14}]. (4) Conclusions: Severe, extensive attachment loss may be indicative of patients with complex cardiac conditions, which underscores the essential role of periodontal status in relation to systemic diseases. The correlations between the rising trends of the inflammatory parameters suggest a potential interconnection between oral and systemic inflammation.

## 1. Introduction

Periodontitis, atherosclerotic cardiovascular disease (ACVD), and atrial fibrillation represent a cluster of chronic conditions instigated by inflammation, imposing significant burdens on populations globally. These conditions result in severe morbidities, varying levels of mortality [1,2,3,4,5,6,7,8], and considerable financial burdens on public health systems [8,9]. In 2019, about 11% of the world’s population was affected by severe periodontitis, totaling approximately 1.1 billion cases, while milder forms impacted up to 50% of the population [10]. Conversely, cardiovascular diseases (CVDs) accounts for around 32% of global fatalities, with Europe alone reporting over 3.9 million deaths in 2017 [2]. Atrial fibrillation is estimated to affect 2–4% of the general population, with prevalence increasing with age [11].

Periodontitis plays a crucial role in the development of atherosclerosis and ACVD by triggering various pathogenic processes, including the release of pro-inflammatory molecules, oxidative stress imbalances, and disruptions in reactive oxygen species (ROS) and nitric oxide (NO) levels [12]. Moreover, periodontitis has been linked to elevated serum levels of cell-free DNA (cfDNA) [12] and impaired endothelial progenitor cell function [13], further exacerbating atherosclerosis progression. Inflammatory pathways act as a critical link between periodontitis and ACVD [14], as well as atrial fibrillation [15]. The substantial bacterial load in periodontal pockets perpetuates systemic inflammation, which is pivotal in initiating and advancing ACVD [14] and its associated complications [16]. This heightened systemic inflammation significantly raises the risk of various cardiovascular events, such as myocardial infarction [17,18], heart failure [19], and stroke [20].

In recent years, several potential molecular and hematologic-cell markers have surfaced to indicate systemic inflammation across various pathologies [21]. Elevated circulating levels of C-reactive protein (CRP) have been consistently noted in both patients with periodontitis [22] and those with ACVD [23]. Increased white blood cell (WBC) counts have been linked to both periodontitis [24] and ACVD [25], including its acute events [26]. CRP and other inflammatory biomarkers such as TNF-α and IL-6 are linked to various aspects of atrial fibrillation, including occurrence, recurrence, burden, and thromboembolic complications [27]. Various pathologies inducing systemic inflammation led to fluctuations in WBC populations and changes in certain hematological indices [28], such as the neutrophil-to-lymphocyte ratio (NLR), eosinophil-to-lymphocyte ratio (ELR), platelet-to-lymphocyte ratio (PLR), lymphocyte-to-monocyte ratio (LMR), or systemic immune-inflammation index (SII) [29]. Associative relationship between NLR, LMR, and PLR and periodontitis have been identified by a recent meta-analysis [30], but no information of this type has been identified when periodontitis patients with systemic diseases such as diabetes, cardiovascular, or autoimmune rheumatic diseases have been considered. This underscores the need and opportunity to conduct research in this area.

Potential indicators of systemic or local inflammation, including S100A8/A9 (calprotectin), interleukin-1 (IL-1), and kallikrein, have been associated with periodontitis [31] as well as ACVD [32,33,34] and atrial fibrillation [15].

Studying the salivary expression of inflammation-related mediators could aid in identifying patients with periodontitis, particularly those at a higher risk for ACVD-related complications. Salivary levels of S100A8/A9 have been linked to periodontal damage and could be considered a reliable prognostic marker for periodontitis [35]. Higher levels of salivary kallikrein, especially kallikrein-13, may suggest the severity of bacterial infection in periodontitis. This increase might result from the breakdown of tissue-specific kallikrein inhibitors, like SPINK6, by gingipains from *Porphyromonas gingivalis* colonizing periodontal lesions [36]. Disruption in kallikrein family molecules might be a potential risk factor for cancer development and progression [36,37]. Moreover, elevated levels of salivary kallikrein could potentially spread systemically, further worsening the progression of ACVD.

Given the established pathogenetic [7,15,38] and epidemiological [2,3,4,5,6,7,15] connections between periodontitis and ACVD and atrial fibrillation, continuous research into their relationship and the impact of periodontitis on systemic inflammation is imperative. There is a need to identify novel and effective minimally invasive and inexpensive prognostic parameters that would enable periodontologists and cardiologists to pinpoint periodontitis patients at higher risk of systemic cardiovascular complications in order to promptly implement tailored therapeutic periodontal approaches for reducing subgingival infection as well as local and systemic inflammation to prevent cardiovascular morbidity.

Given this context, the present study aims to evaluate the potential role of specific hematological indices (NLR, MLR, PLR, ELR, SII) in addition to a classic systemic marker CRP and three potential salivary inflammation-related molecules (IL-1β, S100A8/A9, kallikrein) to predict periodontitis and its severity assessed based on the new European Federation of Periodontology (EFP)/American Academy of Periodontology (AAP) classification criteria [39,40] in subjects with cardiac diseases and to appreciate the relations of the inflammatory burden and periodontal destruction with the type of cardiac pathology.

## 2. Materials and Methods

### 2.1. Study Design

In this prospective cross-sectional study, patients were picked from the Cardiology Department of the Clinical Rehabilitation Hospital in Cluj-Napoca. Ethical approval was obtained from both the hospital (approval number 4048/23 April 2021) and the Iuliu Hațieganu University of Medicine and Pharmacy in Cluj-Napoca (approval number 249/30 June 2021). All patients gave written informed consent voluntarily and, after that, received clinical examinations and were sampled for biological markers. This research took into account the principles of the Declaration of Helsinki for research, including human individuals.

This study was conducted between July 2021 and December 2023, with interruptions during the peaks of the COVID-19 pandemic. The study group comprised individuals diagnosed with two of the most common cardiovascular conditions: ACVD; arrhythmia; or both ACVD and arrhythmia. These subjects underwent comprehensive medical evaluations related to their systemic diseases, an integral periodontal evaluation in a hospital setting. Also, saliva samples were collected to measure three inflammation-related molecules. Medical data were extracted from the patients’ medical records.

To ensure accurate reporting, this paper was prepared following the STROBE (Strengthening the Reporting of Observational Studies in Epidemiology) guidelines [41].

### 2.2. Participants and Inclusion Criteria

The study group consisted of patients with ACVD and/or arrhythmia who were referred from hospitals in the north-central region of the country. Patients were recruited consecutively, two days a week, based on the hospital register. Inclusion criteria were as follows: stable ACVD (including atherosclerotic coronary artery, cerebrovascular, and peripheral vascular diseases), as documented in medical letters (with stable symptoms for at least 60 days), or cardiac rhythm disorders; no recent myocardial damage; absence of severe diseases such as cancer or autoimmune rheumatic diseases and severe immunosuppression; no periodontal therapies in the last year; and having at least 10 teeth (excluding third molars). Exclusion criteria included the following: not meeting the defined criteria for stable ACVD; pregnancy; having undergone systemic antibiotic therapy in the last three months; fewer than 10 teeth (excluding third molars); and suffering from other types of periodontal pathologies or debilitating affections.

### 2.3. Appreciation of Demographic and Systemic Medical Attributes

#### 2.3.1. Demographic Characteristics and Blood Sample

Demographic data, including age, sex, residential location (urban or rural), education level, body mass index (BMI in kg/m^2^), and behaviors (smoking and alcohol consumption), were obtained from the patients’ medical files. On the day of the oral examination, these data were verified for completeness and accuracy, with any discrepancies resolved prior to the examination.

Patients were categorized as smokers/former smokers or non-smokers and as alcohol consumers or non-consumers, according to the criteria established by the National Center for Health Statistics and listed in the Centers for Disease Control (CDC) glossary [42,43].

Blood samples were collected from all participants in the morning following a 12-hour fast. Standard parameters measured included fasting blood glucose, total cholesterol, low-density lipoprotein (LDL) cholesterol, high-density lipoprotein (HDL) cholesterol, triglycerides, CRP, red blood cell count, and leukocyte and platelet counts, using the Sysmex XS-1000i TM (Sysmex Corporation, Osaka, Japan).

#### 2.3.2. Blood-Related Parameters and Inflammatory Blood Cell Indices

Blood parameters collected at baseline were platelets (10^3^/μL), neutrophils (10^3^/μL), lymphocytes (10^3^/μL), monocytes (10^3^/μL), and eosinophils monocytes (10^3^/μL).

We calculated five blood cell-related indices using an MS Excel function: (1) neutrophil to lymphocyte ratio (NLR), calculated as NLR = neutrophils/lymphocytes; (2) platelet to lymphocyte ratio (PLR), calculated as PLR = platelets/lymphocytes; (3) monocyte to lymphocyte ratio (MLR), calculated as MLR = monocytes/lymphocytes; (4) eosinophil/lymphocyte ratio (ELR), calculated as ELR = eosinophil/lymphocyte; (5) systemic inflammation index (SII), calculated as SII = [neutrophils X platelets]/lymphocytes].

The blood sample analyses were performed on Sysmex XS-1000i TM (Sysmex Corporation).

#### 2.3.3. Diagnosis of Cardiovascular Diseases and Other Pathologies

The cardiovascular diagnoses, serving as inclusion criteria in this study, were approved or modified by cardiologists from the Clinical Rehabilitation Hospital Cluj-Napoca (D.P., B.C.) following comprehensive examinations. Patients failing to meet the initial diagnosis criteria were excluded. Clinical examinations, electrocardiogram (ECG) analyses, and cardiac ultrasounds were used for patient evaluations. Patients with palpitations or a history of arrhythmia underwent 24-hour Holter ECG monitoring, while those with typical angina pectoris underwent exercise tests. A positive diagnosis of rhythm disorders (such as atrial fibrillation) was established if arrhythmia was evident on a 12-lead electrocardiogram or persisted for more than 30 s during Holter ECG monitoring. A positive stress test for myocardial ischemia was considered if an ST depression of at least 1 mm in amplitude occurred in two contiguous leads during physical exercise compared to the resting electrocardiogram. Vascular ultrasound was conducted for cases of peripheral arterial disease, with plaques causing at least 50% vascular stenosis deemed clinically relevant. The diagnosis was formulated based on the newest clinical guidelines of the European Society of Cardiology [44,45,46,47,48].

Diagnosis of type 1 and 2 diabetes mellitus or other systemic pathologies was established in accordance with current guidelines, as provided by the medical records.

### 2.4. Assessment of Periodontal Parameters and Case Definitions of Periodontal Conditions

#### 2.4.1. Periodontal Parameters

Experienced investigators previously calibrated (A.C., A.S., I.C.M) [49,50], and one young specialist (C.S.C.) dealt with the periodontal examinations. At the beginning of the research, all investigators received written guidance explaining the study design, periodontal exploration, and data collection protocols. They underwent training for performing the evaluations and were supervised by two senior periodontists (A.R., P.Ș.). Intra- and inter-examiner reproducibility values were 0.95 and 0.94, respectively.

The study group benefited from a comprehensive periodontal assessment for all teeth except wisdom teeth, utilizing established methodologies [49,50,51] and standard examination tools (dental mirror, periodontal probe/University of North Carolina -15 periodontal probe from Hu-Friedy, Chicago, IL, USA). Each tooth was examined at six sites for parameters including probing depth (PD), gingival recession, and clinical attachment level (CAL). Additionally, full-mouth assessments of the gingival bleeding index (GBI) and oral hygiene index (OHI) were conducted according to standardized criteria [52,53]. Tooth mobility, furcation involvement, and missing teeth counts were also documented [50,54].

#### 2.4.2. Case Definition of Periodontal Status

Periodontitis was determined according to the most recent case definition system outlined by the EFP/AAP in 2018 [39,55]. Diagnosis included specific criteria such as the presence of interdental CAL at a minimum of two non-adjacent teeth or buccal/oral CAL ≥3 mm accompanied by PD >3 mm. The periodontal patients were categorized into stages considering the extent of periodontal loss and additional complexity factors, including tooth mobility, posterior bite collapse, deep pockets, and furcation lesions [39,55]. Gingivitis cases were characterized by the absence of CAL and a GBI > 10%. All other cases were considered periodontally healthy. A four-level categorical range was established: (a) stage I (mild) periodontitis; (b) stage II (moderate) periodontitis; (c) stage III plus IV (severe) periodontitis; and (d) gingivitis and periodontally healthy situations.

#### 2.4.3. Saliva Sampling

Saliva samples were collected during the first hours of the day in 50 mL sterile centrifuge tubes (STARLAB International GmbH, Hamburg, Germany). Subjects applied the passive drooling technique [56] to passively accumulate saliva in a collection tube introduced in ice for 15 min.

Subsequently, the samples were promptly frozen at −80 °C until evaluation, adhering to established guides [57].

### 2.5. Quantification of Salivary S100A8/A9, IL-1β, and Kallikrein with Immunoenzymatic Testing (ELISA)

S100A8/A9 (calprotectin L1) IL-1b and human kallikrein-1 levels in the saliva samples were evaluated. Firstly, saliva samples were thawed, aliquoted, centrifuged for 8 min at 220× *g* and 4 °C, and included in ice until the loading stage. For loading, 100 μL of each sample was plated and then treated based on the manufacturers’ specifications.

S100A8/A9 concentration was determined using an ELISA kit (Cat. No. ELH-S100A8-9; RayBiotech, Norcross, GA, USA). ELISA sensitivity value of 0.035 ng/mL was fixed. The sensitivity value was taken into consideration for values under the detection limit.

IL-1β was quantified with an ELISA kit (Cat. No. E0563h; EIAab, Wuhan, China). ELISA sensitivity value of 0.010 ng/mL was considered. Values below the detection limit were replaced with the sensitivity value.

Kallikrein was quantified based on an ELISA kit (Cat. No. E0967h; EIAab, Wuhan, China). ELISA sensitivity value was 0.13 ng/mL. This value was considered for data below the detection limit.

For all assays, the absorbance was appreciated at 450 nm using a TECAN SPARK 10M spectrophotometer (Männedorf, Switzerland).

### 2.6. Statistical Analysis

Categorical data were presented as counts and percentages and compared with chi-squared or Fisher exact tests. Non-normally quantitative data were presented as medians and interquartile ranges and compared with Wilcoxon rank-sum tests in the case of two independent groups or Kruskal–Wallis for three groups (followed by Dunn post-hoc tests). The Spearman correlation coefficient and its corresponding *p*-value were used to assess the correlation between non-normally distributed quantitative variables. For all statistical tests, the two-tailed *p*-value was reported, and a 0.05 value was considered as the level of statistical significance. All statistical analyses were carried out with the R environment for statistical computing and graphics (R Foundation for Statistical Computing, Vienna, Austria), version 4.2.2 [58].

## 3. Results

**Figure 1:**
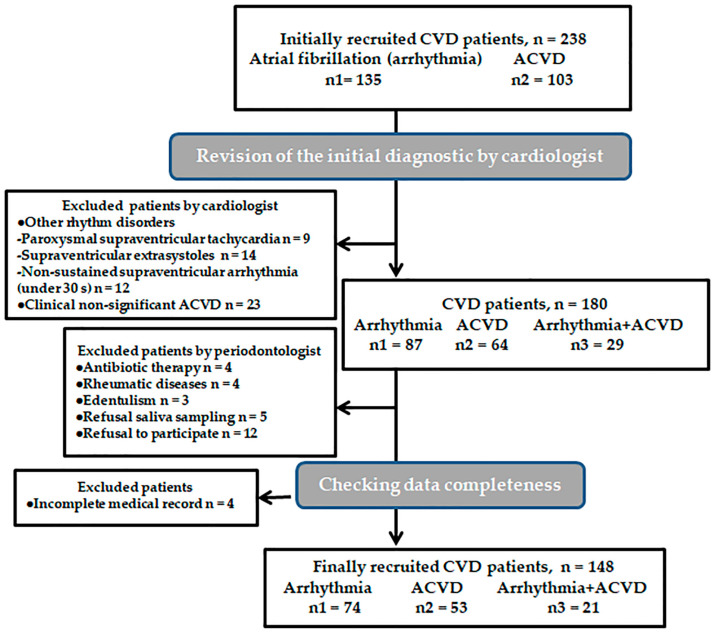
Flowchart of the patients. Abbreviations: ACVD, atherosclerotic cardiovascular disease; CVD, cardiovascular disease; s, seconds; n, number.

A total number of 148 participants with a mean age of 66 were admitted in this cross-sectional study (Figure 1). All patients enrolled in this study exhibited periodontitis, categorized into stage II as well as stage III/IV, indicating the absence of any other periodontal conditions. Based on periodontitis severity, their demographic and behavioral attributes, as well as the systemic pathologies, are shown in Table 1. The severity of periodontitis was associated with the severity of cardiac involvement, equivalent to the presence of both ACVD and arrhythmia (*p* = 0.038).

Considering the two-periodontitis severity scale, periodontal parameters, as well as salivary and systemic biological constants, are indicated in Table 2. Periodontitis severity-related parameters (mean interproximal CAL, number of sites with CAL ≥ 5 mm, mean and median PD, number of sites with PD = 5 and PD > 5) were significantly more elevated in the stage III/IV group of patients than in the stage II group of patients (*p* < 0.001). Statistically significantly increased S100A8/A9 levels (*p* = 0.004) were present in severely affected individuals than in patients displaying moderate periodontal destructions. No other significant differences in biological indicators were highlighted between stage III/IV and stage II patients.

The number of sites with PD = 5 mm correlated with PD values [Spearman correlation coefficient (*p*-value): mean PD values 0.72 (<0.001) and median PD values 0.62 (<0.001)], with interproximal CAL [0.51 (<0.001)] and number of sites with CAL ≥ 5 mm [0.51 (<0.001)], which confirms a similar trend followed by severity-related parameters (Table S1).

GBI correlated with mean and median PD [0.48 (<0.001); 0.43 (<0.001)], the number of sites PD = 5 mm [0.28 (<0.001)], and the number of sites PD > 5 mm [0.3 (<0.001)]; mean CAL [0.41 (<0.001)] and number of sites with CAL ≥ 5 mm [0.2 (0.013)] (Table S1).

A positive correlation between the levels of salivary S100A8/A9 and those of salivary IL-1β [0.35 (<0.001)], kallikrein {0.55 (<0.001)], and serum CRP [0.28 (<0.001)] was observed. Salivary kallikrein levels positively correlated with serum CRP concentrations [0.17 (0.036)] (Table S1).

A positive correlation between NLR, on the one hand, and, on the other hand, the other blood cell-related indicators MLR [0.68 (<0.001)], PLR [0.55 (<0.001)], SII [0.83 (<0.001)], and CRP [0.34 (<0.001)] were observed (Table S1).

Considering the periodontal and inflammatory parameter modifications based on the type of cardiac involvement revealed that patients with complex cardiac involvement represented by arrhythmia plus ACVD had a significantly higher number of sites with CAL ≥ 5 mm [19 (3–30)] compared to individuals with only arrhythmia [9 (3.25–18)] or CVD [5 (1–12)] [0.048♦ {0.162/0.496/0.14}] (Table 3).

## 4. Discussion

Our study explored the relationships between some local (S100A8/A9, IL-1β, kallikrein) and systemic (CRP, NLR, MLR, PLR, ELR, SII) inflammatory markers with the presence and severity of periodontitis in a cohort of cardiovascular patients. Additionally, we examined the relationship between inflammatory burden and parameters associated with periodontal destruction and how they relate to some of the most frequent cardiac diseases.

All cardiac patients in our study exhibited periodontitis, primarily severe forms (stage III and IV), assessed based on the case definition system outlined in the new EFP/AAP classification published in 2018 [39,40]. This finding is somewhat surprising considering the reported overall periodontitis prevalence of 50% in the general population [59]. Conversely, utilizing the same case definition system, our team reported markedly elevated periodontitis frequency rates: 97.8% in stroke patients [49]; 88.68% in systemic sclerosis patients; and 85.85% in those with CVD [51]. Newly available data reveal that among periodontitis patients with CVD, 58% exhibited severe periodontitis [60]. However, they did not adhere to the protocols associated with the new classification system’s criteria for defining periodontitis.

In our cohort of patients, significantly statistically more stage III/IV periodontitis patients exhibit cardiac disease (arrhythmia, ACVD, and arrhythmia plus ACVD) than stage II periodontitis patients (*p* = 0.038) (Table 1). Moreover, patients with complex cardiac involvement (arrhythmia plus ACVD) exhibited a significantly increased number of severely affected sites (CAL ≥ 5 mm) [19 (3–30)] vs. [9 (3.25–18)] and [5 (1–12)] [0.048♦ {0.162/0.496/0.14}], which can reflect a dose-related relationship between periodontitis and cardiac diseases. Periodontitis has been linked to ACVD [7] and its severe forms with an increased risk of cerebrovascular pathology [61]. Furthermore, the presence of periodontitis significantly increases the risk of new-onset atrial fibrillation in a dose-dependent pattern [61,62,63]. Increased concentrations of inflammatory markers like CRP, TNF-α, and IL-6 are linked to atrial fibrillation occurrence, recurrence, and thromboembolic complications [27]. The impact of nonsurgical periodontal treatment on atrial fibrillation outcomes is inconclusive [62,64,65]. However, studies linking periodontitis and atrial fibrillation have limitations, mainly concerning diagnostic criteria and examination protocols [15]. A positive point of our research addresses actual unclarities in reporting periodontitis by using a standardized examination and periodontitis definition system [39,40,55], enhancing the reliability of results and facilitating comparisons across studies. Using the new classification system to report periodontitis in patients with arrhythmia represents a novel aspect of our study.

The significant association observed by our study between the periodontitis stages and the severity of destruction-related parameters (mean CAL and the mean number of severe CAL) (*p* < 0.001) is a logical outcome stemming from the definition of periodontitis cases [40]. Additionally, the significant positive relationship (*p* < 0.001) between periodontitis stages and mean PD underscores PD’s significance as an important complexity factor in characterizing the severity of periodontitis. It is unsurprising that PD cut-off values are incorporated into the therapeutic decision-making process for periodontitis [7].

Among the three salivary inflammation markers examined by our study, only levels of salivary S100A8/A9 were found to be significantly associated with the severity of periodontitis [4.68 ng/mL (2.34–7.31) in the stage II patients versus 7.76 ng/mL (3.8–9.57) in the stage III/IV patients, *p* = 0.004]. Notably, within our patient cohort, a positive correlation was observed between salivary S100A8/A9 and other salivary biomarkers [IL-1β 0.35 (<0.001), kallikrein 0.55 (<0.001)], as well as serum CRP [0.28 (<0.001)]. Also, kallikrein levels correlated with CRP concentrations [0.17 (0.036)]. These correlations indicate a concurrent rise in multiple inflammation-related molecules, suggesting systemic inflammatory dysregulation in individuals affected by both periodontitis and ACVD. Elevated levels of S100A8/A9 have already been reported in both serum [31] and saliva [35] among individuals with periodontitis. Such heightened concentrations may potentially elevate the risk of CVD and its related complications [32,33]. IL-1β has a critical role in the development of periodontitis [66]. The translocation of locally secreted IL-1β may elevate serum IL-1 levels, contributing to the development of CVD through its proatherogenic and procoagulant activities [67]. Although statistically non-significant, our study identified increased levels of salivary kallikrein in stage III/IV periodontitis patients than in stage II. Elevated serum kallikrein constitutes a potential pathogenic risk determinant in the development of CVD and major adverse cardiovascular events [34].

In our study, CRP median values calculated in both moderate (stage II) and severe (stage III/IV) periodontitis [0.4 ng/mL (0.2–1.2) vs. 0.4 (0.2–1), *p* = 0.765] can be considered within the normal range. However, such concentrations have been regarded by others as mildly elevated and have been associated with obesity, pregnancy, diabetes, periodontitis, or a sedentary lifestyle [68]. Moreover, some of our patients presented CRP concentrations over 1 mg/dL, which could place them at cardiovascular risk. It has been reported that in such patients, subgingival mechanical instrumentation induced a greater reduction in CRP values, particularly when baseline CRP levels exceeded 3 mg/L [69]. This insight is particularly noteworthy, as decreasing CRP levels in CVD patients to below 2 mg/mL has been shown to reduce the risk of cardiovascular death by one-third, regardless of other risk factors [70].

Interestingly, our study revealed that the cell-based markers indicative of systemic inflammation exhibited comparable values in patients with stage II periodontitis and those with stage III periodontitis (NLR 1.82 (1.42–2.08) vs.1.87 (1.45–2.75), *p* = 0.153; MLR 0.28 (0.24–0.34) vs. 0.31 (0.25–0.38), *p* = 0.16; PLR 105.94 (85.2–118.95) vs. 106.1 (86.86–133.31), *p* = 0.444; ELR 0.08 (0.06–0.15) vs. 0.09 (0.05–0.12), *p* = 0.411; SII 336.85 (282.12–563.67) vs. 407.26 (302.13–642.33), *p* = 0.176). This could be attributed to the possibility that stage II periodontitis may have already initiated systemic inflammatory changes similar to those induced by more severe stages, making them indistinguishable.

The NLR value of 1.87 (1.45–2.75) observed in our study is lower than the 2.22 (SD ± 0.85) reported in a group of stage III periodontitis patients and is closer to the value recorded in healthy periodontal conditions [1.85 (SD ± 0.67)] [71]. Other reported mean values of NLR include 2.15 (SD 1.5–2.9) in healthy adults [72] and a range of 5–31 in individuals with infectious diseases [28].

The SII value of 407.26 (302.13–642.33) obtained in our group of stage III periodontitis patients is significantly lower than the 802.29 (473.08–1,390.30) reported for a group of CVD patients [73] and lower than the value considered significant for an increased incidence of cardiac death [74]. However, standardized cut-off values delineating different pathologies are not yet available in the literature.

Our findings are opposed to recent data, indicating that periodontitis has a statistically significant relationship with NLR and LMR and a debatable association with PLR [30]. This may be due to the fact that all cardiac disease patients of the present study exhibit periodontitis, predominantly severe forms, leading to significant serum changes associated with systemic inflammation induced by both pathological conditions. However, investigating blood cell-based indices in patients with both periodontitis and CVD and their relationships with other inflammation-related markers can be considered a novel aspect of the present study. In the meantime, identifying the complex interplay between local and systemic inflammation-related parameters, their interdependence, and their quantitative contribution to systemic inflammation proved challenging. Several uncontrollable factors could have influenced the levels of inflammatory markers differently in saliva and plasma, such as interindividual variability (including the severity of systemic conditions, medications, and lifestyles), technical variability (including sample handling, storage conditions, and assay procedures), and marker sensitivity. Standardizing these factors in future research could enhance the accuracy of the associative relationships. The present finding highlights a limitation of this study, namely, the absence of a control group comprising individuals without periodontitis. It also emphasizes the importance of further investigations involving non-periodontitis patients to observe changes in blood cell-based indices as a measure of the systemic inflammatory status. Further limitations are represented by the observational nature of our study, which precludes causality affirmations.

The positive correlation between NLR and each of the other inflammation systemic indicators (MLR, PLR, SII, CRP) [0.34 (< 0.001)] identified by our study sustains that systemic inflammation triggers an extensive deterioration of blood cell counts impacting the modification of respective indices. Fluctuations in circulating WBC populations have been linked with pathological conditions [28]. The NLR and PLR have been identified as valuable biomarkers for indicating systemic inflammation in various conditions, including diabetes [75], inflammatory diseases [76], malignancies [77], periodontitis [30], as well as a range of CVD and their associated complications [26,78,79]. Other hematological indices have also been identified as potential markers for different pathologies, including nasal polyposis and carcinoma, such as ELR [80], PLR, LMR, or SII [29]. Determining the extent to which the increase in blood-cell indices is attributable to periodontitis could facilitate the development and clinical implementation of more rigorous protocols for periodontitis screening and treatment in cardiac disease patients, ultimately reducing the systemic risks associated with dysregulated blood-cell counts.

The enormous periodontitis burden in our study group highly encourages further research in this field in order to provide dental practitioners and cardiologists with easy-to-use tools to identify periodontitis patients at risk of systemic inflammation and related complications. Conducting further studies that include patients with both periodontitis and CVD, as well as well-matched control individuals without periodontitis, will provide insights into the relationships between periodontitis, inflammation-related molecules, and blood cell-related indicators. Enhanced cooperation between oral health practitioners and family physicians has already been highlighted as crucial for the timely identification and treatment of non-communicable diseases such as periodontitis [61].

## 5. Conclusions

Severe, extensive attachment loss may be indicative of patients with complex cardiac conditions, which underscores the essential role of periodontal status in association with systemic diseases.

Salivary S100A8/A9 levels associated with periodontitis severity. A positive correlation between the levels of salivary S100A8/A9 with serum CRP was identified, which incites future research to establish the role of this molecule as a potential severity marker of periodontitis and systemic inflammation.

NLR positively correlated with the other blood cell-related indicators, highlighting that systemic inflammation triggers an extensive deterioration of blood cell counts and the related indices.

### Clinical Relevance

The identification of reliable hematological markers for tracking systemic inflammation induced by periodontitis would allow us to identify patients at risk for CVD adverse effects.

## Figures and Tables

**Table 1:** Demographic, behavioral, and general medical characteristics of the patients in relation to the severity of periodontal destruction.

Characteristics	Periodontitis Staging		*p*-Value
	Stage II (n = 33)	Stage III/IV (n = 115)	
**Demography**			
Gender (F), n (%)	21 (63.64)	54 (46.96)	0.091
Rural/Urban (Rural), n (%)	17 (51.52)	56 (48.7)	0.775
Education level, n (%)			0.098
Academic	5 (15.15)	15 (13.04)	
Highschool	27 (81.82)	80 (69.57)	
Gymnasium	1 (3.03)	20 (17.39)	
**Behaviors**			
Alcohol, n (%)	14 (42.42)	70 (60.87)	0.059
Actual/former smokers, n (%)	5 (15.15)	43 (37.39)	0.016
**General medical characteristics**			
Cardiac disease n (%)			0.038
Arrhythmia	12 (36.36)	62 (53.91)	
ACVD	18 (54.55)	35 (30.43)	
Arrhythmia + ACVD	3 (9.09)	18 (15.65)	
Diabetes, n (%)	10 (30.3)	22 (19.13)	0.169
Dyslipidaemia, n (%)	15 (45.45)	47 (41.23)	0.665
Obesity, n (%)	11 (33.33)	32 (27.83)	0.539

Abbreviations: ACVD, atherosclerotic cardiovascular disease; n, number.

**Table 2:** Periodontal parameters and local and systemic inflammatory indicators of the patients in relation to the severity of periodontal destruction.

Characteristics	Periodontitis Staging		*p*-Value
	Stage II (n = 33)	Stage III/IV (n = 115)	
**Periodontal parameters**			
Examined teeth, median (IQR)	12 (6–22)	14 (6.5–18)	0.947
Absent teeth, median (IQR)	14 (6–22)	14 (10–21.5)	0.791
Total probed sites, median (IQR)	114 (60–138)	78 (42–108)	0.011
IHI%, median (IQR)	0.47 (0.32–0.75)	0.55 (0.36–0.8)	0.348
GBI%, median (IQR)	0.25 (0.14–0.53)	0.31 (0.19–0.52)	0.267
Mean CAL_interproximal_, median (IQR)	2.2 (0.8–2.62)	3.45 (2.71–4.39)	<0.001
No sites with CAL ≥ 5 mm, median (IQR)	0 (0–1)	11 (5.5–20.5)	<0.001
Mean PD, median (IQR)	1.71 (1.45–2.33)	2.54 (2.05–3.26)	<0.001
Median PD, median (IQR)	2 (1–2)	2 (2–3)	<0.001
No sites with PD = 5, median (IQR)	0 (0–1)	6 (2–12.5)	<0.001
No sites with PD > 5, median (IQR)	0 (0–0)	1 (0–3)	<0.001
**Local (salivary) inflammation markers**			
S100A8/A9 (ng/mL), median (IQR)	4.68 (2.34–7.31)	7.76 (3.8–9.57)	0.004
IL-1β (ng/mL), median (IQR)	0.01 (0.01–0.02)	0.01 (0.01–0.03)	0.079
Kallikrein (ng/mL), median (IQR)	0.17 (0.13–0.39)	0.33 (0.13–0.53)	0.139
**Systemic inflammation indicators**			
CRP, median (IQR)	0.4 (0.2–1.2)	0.4 (0.2–1)	0.765
NLR, median (IQR)	1.82 (1.42–2.08)	1.87 (1.45–2.75)	0.153
MLR, median (IQR)	0.28 (0.24–0.34)	0.31 (0.25–0.38)	0.16
PLR, median (IQR)	105.94 (85.2–118.95)	106.1 (86.86–133.31)	0.444
ELR, median (IQR)	0.08 (0.06–0.15)	0.09 (0.05–0.12)	0.411
SII, median (IQR)	336.85 (282.12–563.67)	407.26 (302.13–642.33)	0.176

Abbreviations: IHI, hygiene index; GBI, gingival bleeding index; CAL, clinical attachment loss; PD, probing depth; IL-1β, interleukin-1β; CRP, C-reactive protein; S100A8/A9 (calprotectin), NLR, neutrophil/lymphocyte ratio; MLR, monocyte/lymphocyte ratio; PLR, platelet/lymphocyte ratio; ELR, eosinophil/lymphocyte ratio; SII, systemic inflammation index; IQR, interquartile range; n, number.

**Table 3:** Relationships of periodontal parameters with the severity of cardiac involvement.

Cardiovascular Diseases	Arrhythmia (n = 74)	ACVD (n = 53)	Arrhythmia + ACVD (n = 21)	*p*{(1,2)/(1,3)/(2,3)}
Mean PD, median (IQR)	2.39 (1.81–2.87)	2.42 (1.9–3)	2.44 (2.09–3.21)	0.601♦ {0.761/0.593/0.977}
Median PD, median (IQR)	2 (2–2.5)	2 (2–3)	2 (2–3)	0.355♦ {0.781/0.287/0.623}
No sites with PD = 5, median (IQR)	5 (0.25–9)	3 (0–9)	3 (0–10)	0.7♦ {0.681/1/0.877}
No sites with PD > 5, median (IQR)	0 (0–2)	0 (0–2)	0 (0–1)	0.736♦ {0.932/0.854/0.724}
S100A8/A9(ng/mL), median (IQR)	7.19 (3.57–9.66)	4.81 (3.45–8.17)	7.93 (4.98–8.78)	0.236♦ {0.344/0.995/0.225}
IL-1β (ng/mL), median (IQR)	0.01 (0.01–0.02)	0.01 (0.01–0.04)	0.02 (0.01–0.04)	0.104♦ {0.576/0.097/0.473}
Kallikrein (ng/mL), median (IQR)	0.32 (0.13–0.48)	0.25 (0.13–0.52)	0.37 (0.2–0.52)	0.523♦ {0.846/0.776/0.436}
GBI%, median (IQR)	0.25 (0.18–0.52)	0.31 (0.15–0.53)	0.3 (0.23–0.46)	0.77♦ {0.89/0.748/1}
Mean CAL_interproximal_, median (IQR)	3.1 (2.46–4.06)	3.09 (2.22–4.17)	3.65 (2.89–4.47)	0.19♦ {0.985/0.209/0.163}
No sites with CAL ≥ 5 mm, median (IQR)	9 (3.25–18)	5 (1–12)	19 (3–30)	0.048♦ {0.162/0.496/0.14}

Abbreviations: ACVD, atherosclerotic cardiovascular disease; IL-1β, interleukin-1 β; GBI, gingival bleeding index; CAL, clinical attachment loss; PD, probing depth; IQR, interquartile range; n, number.

## Data Availability

The datasets generated during the current study are available from the corresponding author upon reasonable request.

## Supplementary Materials

The following supporting information can be downloaded at: https://www.mdpi.com/article/10.3390/biomedicines12061341/s1, Table S1. Correlations between periodontal and inflammatory parameters in cardiovascular disease patients.
