# Supplementary material for: Exploring Periodontal Conditions, Salivary Markers, and Systemic Inflammation in Patients with Cardiovascular Diseases
Source: Biomedicines. 2024 Jun 17;12(6):1341. doi: 10.3390/biomedicines12061341 (PMC11201987; doi:10.3390/biomedicines12061341)
Supplement: Supplementary file 1 [file biomedicines-12-01341-s001.zip › biomedicines-3039392-supplementary.pdf]

Caloian CS et al. **Exploring Periodontal Conditions, Salivary Markers, and Systemic Inflammation in Patients with Cardiovascular Diseases**

Supplementary Table S1. Correlations between periodontal and inflammatory parameters in cardiovascular disease patients

Periodontal and inflammation-related parameters	Mean PD	Median PD	No sites with PD=5	No sites with PD>5	S100A8/A9 (ng/mL)	IL-1 β (ng/mL)	GBI%	Kallikrein (ng/mL)	Mean CAL interprox	MLR	NLR	No sites with CAL ≥5mm	PLR	SII	CRP
Mean PD	-	0.92 (< 0.001)	0.72 (< 0.001)	0.65 (< 0.001)	0.08 (0.315)	0.05 (0.548)	0.48 (< 0.001)	-0.07 (0.369)	0.72 (< 0.001)	-0.01 (0.869)	-0.04 (0.608)	0.6 (< 0.001)	0.03 (0.73)	0.05 (0.556)	0 (0.986)
Median PD	0.92 (< 0.001)	-	0.62 (< 0.001)	0.57 (< 0.001)	0.1 (0.243)	0.01 (0.867)	0.43 (< 0.001)	-0.07 (0.382)	0.66 (< 0.001)	-0.04 (0.617)	-0.06 (0.467)	0.52 (< 0.001)	-0.02 (0.802)	0.04 (0.669)	0.01 (0.875)
No sites with PD = 5	0.72 (< 0.001)	0.62 (< 0.001)	-	0.61 (< 0.001)	0.05 (0.528)	0.1 (0.225)	0.28 (< 0.001)	-0.1 (0.211)	0.51 (< 0.001)	-0.04 (0.631)	0 (0.956)	0.73 (< 0.001)	0.12 (0.151)	0.09 (0.255)	-0.06 (0.436)
No sites with PD > 5	0.65 (< 0.001)	0.57 (< 0.001)	0.61 (< 0.001)	-	0.1 (0.215)	0.14 (0.081)	0.3 (< 0.001)	-0.04 (0.607)	0.44 (< 0.001)	-0.16 (0.045)	-0.06 (0.442)	0.51 (< 0.001)	-0.03 (0.679)	0.02 (0.844)	-0.09 (0.271)
S100A8/A9 (ng/mL)	0.08 (0.315)	0.1 (0.243)	0.05 (0.528)	0.1 (0.215)	-	0.35 (< 0.001)	-0.09 (0.284)	0.55 (< 0.001)	0.12 (0.151)	0.09 (0.253)	0.14 (0.08)	0.11 (0.19)	-0.04 (0.614)	0.13 (0.123)	0.28 (< 0.001)
IL-1 β (ng/mL)	0.05 (0.548)	0.01 (0.867)	0.1 (0.225)	0.14 (0.081)	0.35 (< 0.001)	-	0.04 (0.615)	0.64 (< 0.001)	0.03 (0.706)	-0.05 (0.573)	0.05 (0.586)	0.05 (0.52)	-0.04 (0.618)	0.07 (0.422)	-0.01 (0.945)
GBI %	0.48 (< 0.001)	0.43 (< 0.001)	0.28 (< 0.001)	0.3 (< 0.001)	-0.09 (0.284)	0.04 (0.615)	-	-0.09 (0.28)	0.41 (< 0.001)	0.06 (0.477)	0.02 (0.823)	0.2 (0.013)	0.08 (0.362)	0.07 (0.43)	-0.01 (0.95)
Kallikrein (ng/mL)	-0.07 (0.369)	-0.07 (0.382)	-0.1 (0.211)	-0.04 (0.607)	0.55 (< 0.001)	0.64 (< 0.001)	-0.09 (0.28)	-	0 (0.996)	0.08 (0.36)	0.08 (0.322)	-0.03 (0.68)	-0.05 (0.552)	0.02 (0.836)	0.17 (0.036)
Mean CAL interprox	0.72 (< 0.001)	0.66 (< 0.001)	0.51 (< 0.001)	0.44 (< 0.001)	0.12 (0.151)	0.03 (0.706)	0.41 (< 0.001)	0 (0.996)	-	0.07 (0.379)	-0.02 (0.823)	0.78 (< 0.001)	0 (0.976)	0.05 (0.578)	-0.04 (0.645)
MLR	-0.01 (0.869)	-0.04 (0.617)	-0.04 (0.631)	-0.16 (0.045)	0.09 (0.253)	-0.05 (0.573)	0.06 (0.477)	0.08 (0.36)	0.07 (0.379)	-	0.68 (< 0.001)	0.05 (0.563)	0.5 (< 0.001)	0.53 (< 0.001)	0.32 (< 0.001)
NLR	-0.04 (0.608)	-0.06 (0.467)	0 (0.956)	-0.06 (0.442)	0.14 (0.08)	0.05 (0.586)	0.02 (0.823)	0.08 (0.322)	-0.02 (0.823)	0.68 (< 0.001)	-	0.01 (0.938)	0.55 (< 0.001)	0.83 (< 0.001)	0.34 (< 0.001)
No sites with CAL≥5mm	0.6 (< 0.001)	0.52 (< 0.001)	0.73 (< 0.001)	0.51 (< 0.001)	0.11 (0.19)	0.05 (0.52)	0.2 (0.013)	-0.03 (0.68)	0.78 (< 0.001)	0.05 (0.563)	0.01 (0.938)	-	0.03 (0.735)	0.07 (0.404)	-0.08 (0.353)
PLR	0.03 (0.73)	-0.02 (0.802)	0.12 (0.151)	-0.03 (0.679)	-0.04 (0.614)	-0.04 (0.618)	0.08 (0.362)	-0.05 (0.552)	0 (0.976)	0.5 (< 0.001)	0.55 (< 0.001)	0.03 (0.735)	-	0.67 (< 0.001)	0.14 (0.099)
SII	0.05 (0.556)	0.04 (0.669)	0.09 (0.255)	0.02 (0.844)	0.13 (0.123)	0.07 (0.422)	0.07 (0.43)	0.02 (0.836)	0.05 (0.578)	0.53 (< 0.001)	0.83 (< 0.001)	0.07 (0.404)	0.67 (< 0.001)	-	0.29 (< 0.001)
CRP	0 (0.986)	0.01 (0.875)	-0.06 (0.436)	-0.09 (0.271)	0.28 (< 0.001)	-0.01 (0.945)	-0.01 (0.95)	0.17 (0.036)	-0.04 (0.645)	0.32 (< 0.001)	0.34 (< 0.001)	-0.08 (0.353)	0.14 (0.099)	0.29 (< 0.001)	-

Abbreviations: IHI, hygiene index; GBI, gingival bleeding index; CAL, clinical attachment loss; PD, probing depth; IL-1 β, interleukin-1β; CRP, C-reactive protein; S100A8/A9 (calprotectin), NLR, neutrophil/lymphocyte ratio; MLR, monocyte/lymphocyte ratio; PLR, platelet/lymphocyte ratio; ELR, eosinophil/lymphocyte ratio; SII, systemic inflammation index; IQR, interquartile range; n, number.
